# Caveolin-1 as a pathophysiological factor and target in psoriasis

**DOI:** 10.1038/s41514-019-0034-x

**Published:** 2019-02-05

**Authors:** Ilja L. Kruglikov, Philipp E. Scherer

**Affiliations:** 1 Scientific Department, Wellcomet GmbH, Karlsruhe, Germany; 2 Touchstone Diabetes Center, Department of Internal Medicine, University of Texas Southwestern Medical Center, Dallas, TX 75390-8549 USA

## Abstract

Low expression of caveolin-1 (Cav-1) is typical in psoriatic lesions and overexpression of Cav-1 leads to a reduction of inflammation and suppression of epidermal hyperproliferation, thus ameliorating these two well-known hallmarks of psoriasis. At the same time, the interfacial layers of the white adipose tissue (WAT) adjacent to psoriatic lesions demonstrate much higher stiffness, which also points to a modification of Cav-1 expression in this tissue. These processes are connected with each other and regulated via exosomal exchange. Here we discuss the role of Cav-1 expression in inflammatory and hyperproliferative processes and analyze the ways to provide spatially different modulation of Cav-1 expression in the skin and WAT. Such modulation can be induced by different pharmacological and physical factors. These include application of mechanical stress and supra-physiological temperatures. Cav-1 should therefore be considered as an important target in treatment of psoriasis.

## Introduction

Caveolae are characteristic plasma membrane invaginations forming nanodomains with a typical size of 50–100 nm which are found in many different types of cells.^1^ These structures are highly abundant in mechanically stressed cells, such as endothelial cells, fibroblasts, adipocytes and muscle cells, where they constitute up to 50% of the total surface area and can exist as single invaginations or clusters. They are involved in rapid adaptation to cellular volume changes, in different signal transduction processes, as well as in the processes of endocytosis and exocytosis.^2^

As shown for adipocytes under physiological conditions, the surface density of caveolae in cells from a given tissue area appears to be almost constant and independent of the cell size.^3^ The density of caveolae can, however, be dramatically modulated in a dose-dependent manner upon application of osmotic shock or mechanical stress, and this response develops rapidly within minutes upon exerting the physical stress, highlighting the importance of these structures for the adaptation to an altered cellular microenvironment.^2^

Caveolae are enriched in cholesterol and sphingolipids and can contain significant amounts of proteins such as cavins and different types of caveolins (Cav’s). The presence of caveolin-1 (Cav-1), which is the principal structural component of caveolae in the plasma membrane, is necessary for the appearance of caveolae.^4^ Cav-1 protein is not exclusively localized to these microdomains, but is also found in other places within cells, from intercellular junctions to the Golgi complex as well as in mitochondria.

Cav-1 is strongly involved in processes of proliferation and inflammation. Hyperproliferation of epithelial cells and skin inflammation are typical in psoriasis, which is one of the most widespread chronic inflammatory diseases of the skin. Cav-1 is involved in secretion of lamellar bodies, formation of junctions in epidermal keratinocytes and terminal differentiation of these cells.^5^ Decreased expression of this protein was found to be typical in psoriatic lesions^6–9^ as well as in some other hyperproliferative skin disorders.^9^ The level of Cav-1 expression was found to be related to the clinical severity of psoriasis.^7,9^ Moreover, enhanced expression of Cav-1 through administration of Cav-1 scaffolding domain peptide which is a mimetic of the Cav-1 function provided substantial improvement of psoriasiform dermatitis in mice by regulating the cytokine network, including TNF-α.^8^ Cav-1 is therefore not only a marker, but also a target for the treatment of psoriasis.

Recently, we have argued that the superficial layer of the adipose tissue known as dermal white adipose tissue (dWAT) plays an important role in the pathophysiology of psoriasis and that inflammation is typical for the dWAT areas adjacent to the skin lesions.^10,11^ Sonoelastography reveals that WAT located underneath psoriatic lesions is structurally distinctly modified, has a much higher stiffness than the neighboring WAT areas covered by unaffected skin. Therapeutic improvements of the skin lesions significantly correlate with normalization of the WAT structure and its mechanical properties.^12,13^ Increasing stiffness of the adipose tissue can be connected with adipogenic differentiation leading to accumulation of lipid droplets in adipocytes,^14^ with additional expression of intercellular junctions between the cells^15^ as well as with increased collagen production in WAT. Actually, it is not known which of these processes mainly contribute to WAT modification described in ref. ^12^ At the same time, very recently, we found that Cav-1 can be redistributed within adipose tissue via mechanism of exosomal exchange over a long-range.^16^ This mechanism should also be involved in redistribution of Cav-1 between the skin and WAT.

Here we re-consider the pathogenesis of psoriasis with a special emphasis on defective Cav-1 expression/distribution in psoriatic skin and analyze whether physical factors which are known to modulate these processes can be applied for treatment of this skin disease.

## Involvement of Cav-1 in epithelial hyperproliferation and inflammation

### Local correlation of Cav-1 with collagen expression

Cav-1 is involved in control of the cell motility through interactions with the cell cytoskeleton on the inside and with extracellular matrix (ECM) on the outside.^17^ Cav-1 demonstrates a negative correlation with the expression of collagen I, which is especially pronounced in scleroderma,^18^ keloids,^19^ hypertrophic scars,^20^ and chronologically aged skin.^21^ Also, Cav-1 scaffolding domain peptide was shown to demonstrate antifibrotic properties both in vitro and in vivo.^22^

Whereas the surface density of caveolae in adipocytes was found to decrease with the age of subjects,^3^ mRNA expression of Cav-1 was shown to be significantly increased in aged skin. Such behavior of Cav-1 strongly correlates with the reduction of Col1 expression in chronologically aged skin.^21^ Moreover, the suppression of Cav-1 expression in this study caused pronounced up-regulation of Col1 expression in the skin without resulting in skin fibrosis. This correlation is additionally supported by the fact that Cav-1 deficiency leads to enhanced cell death and fibrosis in WAT.^23^

The negative correlation between Cav-1 and collagen expression is also evident in psoriasis where the low levels of Cav-1^6,9^ negatively correlate with high levels of collagen expression in involved and even in uninvolved areas of the skin.^24,25^

### Cooperation of Cav-1 with matrix metalloproteinases (MMPs)

Remodeling of the ECM takes place through complex interactions of Cav-1 with MMPs.^26^ Among them, Cav-1 modulates the expression of gelatinases MMP-2 and MMP-9,^27^ which are very much involved in development of different skin efflorescences.^28^ Suppression of Cav-1 leads to activation of MMP-2/MMP-9 expression; alternatively, induction of Cav-1 causes suppression of these MMPs.^29^ This effect may be related to the fact that MMP-2 and MT1-MMP (MMP14) are co-localized with Cav-1 on the cell surface.^30^ Cav-1 may also be involved in the regulation of some intercellular junctions controlling their degradation by MMP-9.^31^ Additionally, Cav-1 inhibits MMP-1 gene expression in human dermal fibroblasts,^32^ and Cav-1^−/−^ skin fibroblasts have decreased expression of MMP-3.^33^

Lesional scales in psoriasis demonstrate a specific profile of MMPs. Significant overexpression of MMP-2/MMP-9 in early psoriatic plaques was described in ref. ^34^ Whereas no correlation between MMP-2 and psoriasis area and severity index (PASI) was found in lesional scales, MMP-9 increased with PASI, and clinical improvement of psoriatic plaques correlated with a significant reduction of MMP-9 expression in these lesions.^35^ Such temporal and severity-dependent behaviors of gelatinases correlate with reduced Cav-1 expression in psoriatic lesions,^6,9^ along with pronounced dependence of Cav-1 expression on PASI,^7,9^ as well as with a significant increase of Cav-1 expression in healed psoriatic skin.^8^

### Cav-1 in cell cycle regulation and epithelial hyperplasia

Cav-1 is also known as a negative regulator of cell proliferation. This effect was observed in different cell lines where the overexpression of Cav-1 caused cell cycle arrest in the G0/G1 phase.^36,37^ On the other hand, the genetic ablation of Cav-1 stimulates cell proliferation and can even lead to the development of extensive epithelial hyperplasia.^38^ This effect can be connected with the fact that Cav-1 is involved in the formation of junctions in epidermal keratinocytes and terminal differentiation of these cells.^5^

Caveolins are also involved in the generation of gap junctions, and the relative levels of these junctions influence the adhesion strength between the cells.^39^ Since skin aging is strongly dependent on the adhesion between different skin layers as well as between dermis and subcutis,^40,41^ this may be an important mechanism by which caveolins are involved in the aging process.

### Cav-1 in inflammation

There are different indications suggesting that Cav-1 is directly and indirectly involved in tissue inflammation. Glucocorticoids are well-known anti-inflammatory agents which act through binding to glucocorticoid receptors. These receptors are colocalized with Cav-1 and actively interact with this protein.^42^ Both the disruption of caveolae as well as the down-regulation or ablation of Cav-1 lead to an impaired functional impact of glucocorticoids. Expression of glucocorticoid receptors is indeed significantly reduced in psoriatic lesions comparing with normal and non-lesion skin. Additionally, Cav-1 can suppress inflammation through inhibition of endothelial NO synthase (eNOS) activity.^43^ Further, elimination of Cav-1 promotes the polarization of M2 macrophages in mice.^44^ Also, cells deficient in Cav-1 demonstrate a significantly increased uptake of *S. aureus*^45^ which is believed to be involved in psoriasis.^10^ This effect was connected with a negative regulation of the membrane microdomain mobility through Cav-1 which anchors these domains onto the cytoskeleton. Moreover, enhancing membrane fluidity increases the uptake of *S. aureus* by non-professional phagocytic cells. Such enhancement can be provided by different chemical and/or physical factors, including the application of supra-physiological temperatures. Infection of fibroblasts with *S. aureus* at different temperatures (33–41 °C) indeed demonstrated that enhanced mobility of membrane microdomains in these cells strongly correlates with increased bacterial uptake.^45^

Altogether, Cav-1 is directly and indirectly involved in the production of local hyperproliferative conditions and inflammation in psoriatic skin.

### Cav-1 expression in immature and mature adipocytes

Superficial WAT underneath psoriatic lesions is structurally modified.^10–14^ This modification should be related to the Cav-1 expression profile in the skin. How can the cellular content of this adipose layer influence the Cav-1 expression profile? Cav-1 is differentially expressed in immature and mature adipocytes. Cav-1 expression dramatically increases during adipogenesis with a much higher level in mature adipocytes than in precursors.^46,47^ At the same time, the correlations between cell differentiation and Cav-1 expression appears to be reciprocal: Cav-1 deficient mice have an increased mammary stem cell population.^48^ Additionally, stimulated Cav-1 expression can regulate stem cell proliferation and differentiation.^49^

Therefore, the local reduction of Cav-1 content is connected with a shift in the ratio of immature and mature adipocytes in favor of immature cells. Vice versa, local stimulation of Cav-1 expression should increase cell differentiation, thereby increasing the number of mature cells in the tissue.

## Cav-1 and skin morphology

The modulation of cellular Cav-1 content can significantly modify skin morphology. The skin of Cav-1^−/−^ mice displays increased collagen density and significantly higher tensile strength and elastic modulus than the skin of the wild-type animals.^18^ At the same time, these knockout mice demonstrate a significant shift in the balance of synthesis and degradation of collagens towards net synthesis, a phenomenon likely linked to an increased number of local myofibroblasts in the skin. The dermis of Cav-1^−/−^ mice is strongly infiltrated with macrophages and autophagic cells.^18^ Reduced Cav-1 expression is accompanied by a local loss of E-cadherin and β-catenin and a disruption of the epithelial barrier function.^50^ Moreover, Cav-1^−/−^ mice have almost no dWAT layer, both in males and females.^51^ Since this layer is strongly involved in the innate immune response^52^ and in inflammatory responses,^53–55^ any structural modification of dWAT can lead to increased skin susceptibility for inflammation.

All these findings reflect the fact that Cav-1 is involved in the development and regulation of mechanical properties of the skin.

## Adipose and endothelial tissues can interact through Cav-1

Adipose and endothelial tissues can interact via Cav-1. This effect was first demonstrated for perivascular adipose tissue which was able to inhibit endothelial function in the aorta.^56^ Perivascular adipose tissue is able to affect the eNOS expression and activity (and thus modulate the inflammation), inducing enhanced Cav-1 expression in endothelial cells. This finding even led to the formulation of a new anti-atherosclerotic treatment strategy based on the reduction of the inflammation in WAT.^57^ In,^56^ local adipose tissue was demonstrated to be able to increase the expression of Cav-1 in the neighboring endothelial tissue. This suggests an intense crosstalk between the two cell types including a direct exchange of cellular contents.

Whereas the outright mechanism(s) in place for these cellular interactions remain to be better defined, very recent results demonstrate that exosomal exchange, including transfer of Cav-1, is involved.^16^ Despite effective ablation of the Cav-1 gene in adipocytes of Cav-1-KO mice, Cav-1 protein is abundant in these cells. This paradoxical effect was connected with the exchange of Cav-1 loaded exosomes between adipocytes and neighboring endothelial cells.

## Interaction between miRs and Cav-1

Human psoriatic skin has a specific microRNA (miR) profile, with miR-21 (epidermal lesions), miR-31 (psoriatic keratinocytes), miR-146a (psoriatic lesions), and miR-203 (psoriatic lesions) strongly upregulated.^58^ Different groups have shown that miRs can directly target Cav-1. For example, miR-21 induces myofibroblast differentiation,^59^ a phenomenon typical for strongly reduced Cav-1 levels in the skin.^18^ Elevated levels of miR-31, also known as a regulator of fibrogenesis,^60^ were reported to correlate with low levels of Cav-1 at least in some cells.^61^ Additionally, miR-31 is able to suppress autophagy,^62^ which, on the other hand, is increased by suppression of Cav-1.^18^ This is consistent with other reports that implicate Cav-1 as a critical determinant of autophagy.^63^

In addition, miR-146a is a well-known mediator of inflammation which is activated by pathogenic immune stimulation. miR-146a serves as molecular regulator of macrophage polarization and is highly expressed in M2 but not in M1 macrophages.^64^ Macrophages are cells with high plasticity which can undergo a transition between two relatively loosely defined states, the M1 and M2 phenotypes. Macrophages of the M1 subtype secrete mediators which promote inflammation, whereas M2-type macrophages suppress inflammation and promote fibrosis. A reduction of miR-146a promotes M1 and diminishes M2 macrophage polarization.^64^ On the other hand, a deletion of Cav-1 promotes M2 macrophage polarization in mice,^44^ which suggests a possible connection between miR-146a and Cav-1. Finally, Cav-1 is a direct target for miR-203, at least during caloric restriction.^65^

Additionally, miR-26b-5p is strongly upregulated in sWAT underneath the lesional psoriatic skin.^66^ This upregulation can be connected with the processes of adipocyte differentiation^67^ and WAT inflammation.^68^

Taken together, there is a strong cooperation between miRs and Cav-1, with both of these involved in spatial propagation and distribution of hyperproliferative and inflammatory conditions in the skin.

## Vesicular transport of miRs and Cav-1 as a long-range communication mechanism in the skin

Extracellular transport in the tissue is provided by extracellular vesicles which can appear in the form of microvesicles, exosome-like vesicles, exosomes, and membrane particles.^69^ Every type of vesicle is characterized by specific protein markers.^70^

MiRs can be transported in extracellular vesicles, providing spatially long-range intercellular communication and altering the transcriptome of the recipient cells.^71^ For example, fibroblasts are known to secret exosomes enriched in miR-21.^72^ The release of exosomes is connected with the Cav-1-induced suppression of autophagy.^73,74^ Moreover, adipose-derived miRs are able to regulate gene expression in other tissues.^75^

Cav-1 is also transported by extracellular vesicles. Exosomes secreted by prostate cancer^76^ and melanoma cells^77^ contain high levels of Cav-1. It was also shown to be secreted from osteoblasts in the form of matrix vesicles.^78^ Several authors reported that exosomes secreted by endothelial cells also contain high levels of Cav-1^4,79^ Recent results confirmed that small extracellular vesicles are loaded with Cav-1 and some other proteins and lipids capable to modulate cellular signaling and physiology.^16^

## Spatial distribution of Cav-1 in psoriasis

**Fig. 1:**
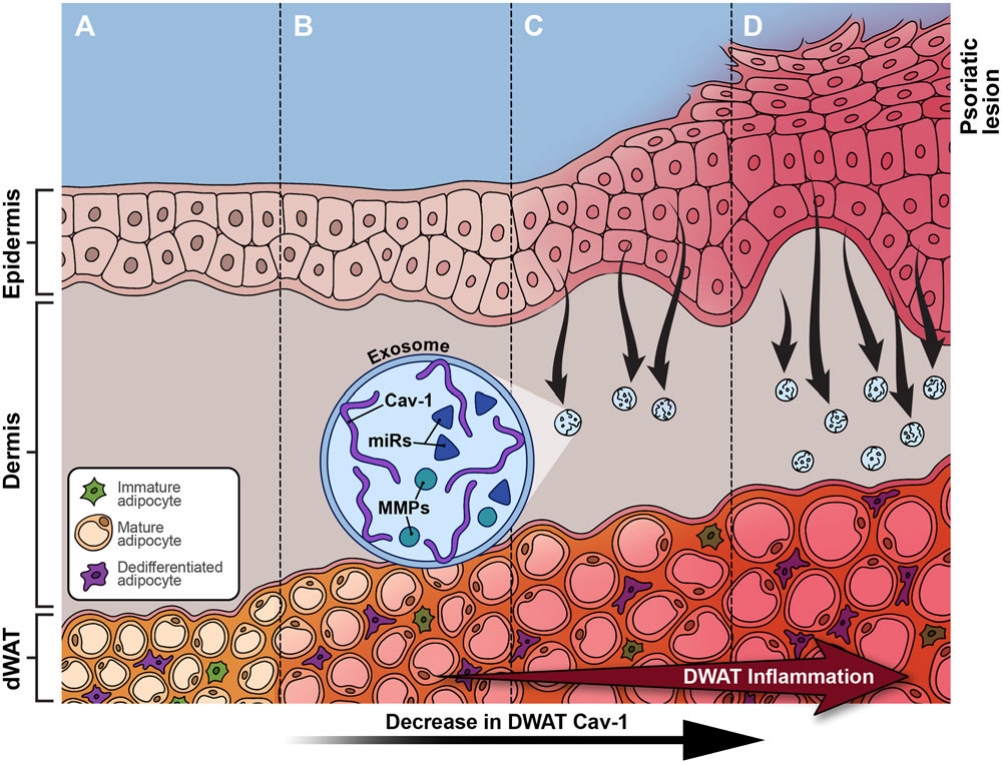
Spatiotemporal interaction of the skin and underlying dermal WAT (dWAT) in psoriasis: **a** normal non-psoriatic skin; **b** modification of the dWAT located beneath the future psoriatic lesion leading to a stable local reduction of Cav-1 content in this tissue and inflammation in this area (Cav-1 expression in the covering skin is normal); **c** to compensate this Cav-1 reduction in dWAT, exosomes start to transport Cav-1 from the skin to dWAT which appears as a sink for Cav-1; **d** this exosomal transport (outflow) is not able to compensate for the strong deficiency of Cav-1 in dWAT; instead, it causes continuous reduction of Cav-1 in the skin and, consequently, its inflammatory state and hyperproliferation

From above, it can be proposed how spatiotemporal behavior of Cav-1 in the skin and the underlying WAT can be involved in the formation of psoriasis. This interaction should include several steps (Fig. 1).

*Step 1*. Inflammation of the superficial WAT located beneath the future psoriatic lesion leading to a stable local reduction of Cav-1 content in this tissue. At this moment, Cav-1 expression in the covering skin is normal.

*Step 2*. To compensate this reduction, exosomes start to transport Cav-1 from the skin to WAT (that means, the skin works as a source and superficial WAT as a sink for Cav-1).

*Step 3*. This exosomal transport (outflow) is not able to compensate the strong Cav-1 deficiency in dWAT. Instead of this, it causes continuous reduction of Cav-1 in the skin and, consequently, its inflammation and hyperproliferation.

## Influence of hyaluronan on transport of miRs and Cav-1

Hyaluronan (HA) is an important component of the skin, which significantly determines its structural and physiological state. Beyond absolute amounts, the molecular weight of HA is crucial for its function: whereas high molecular weight (HMW) HA (>1 MDa) is typical for intact tissue and demonstrates protective effects, low molecular weight (LMW) HA (<3 kDa) induces inflammation.^80^ Content and molecular weight of HA can also significantly modulate the properties of the adipose tissue: whereas HMW HA may promote adipogenesis, LMW HA generated during HA degradation suppresses adipogenesis and induces chemokine expression in macrophages and endothelial cells.^81^ The true physiological relevance of these effects will however need to be validated in vivo.

HA primarily binds to the cell surface through CD44 receptor regulated by MT1-MMP and spatially connected with caveolae.^82^ HA binding is regulated by Col1, demonstrating a negative correlation with Cav-1, and the induced disruption of caveolae provides a tremendous increase of HA binding capacity.^82^

Recently, it was reported that the content and release rate of extracellular vesicles in endothelial cells is dependent on the existence and subtype of hyaluronan present in the media. Long-term (6–24 h) culturing of endothelial cells either without HA, with HMW HA or LMW HA, led to a very differential release of vesicles from these cells.^83^ Both HMW HA and LMW HA significantly increased the release of vesicles containing Cav-1, and this release was spatially connected with caveolae. At the same time, the molecular weight of HA had a strong impact on content and form of released vesicles. Whereas vesicles released by application of LMW HA were characterized as exosomes, vesicles released by application of HMW HA (enlargeosomes) had bigger size, different surface structure, and contained desmoyokin and annexin II.

Whereas the results presented in^83^ were obtained in endothelial cells, we can assume that this effect can also be observed in other cell types. Consequently, exosomes released in psoriatic skin may contain large amounts of miRs and Cav-1 and thus significantly modify the normal communication axis between cells.

## Some ways to modulate Cav-1

Based on the observations discussed above, the manipulation of Cav-1 protein content, release/uptake and transport in psoriatic skin may be an important approach to reduce hyperproliferation and inflammation, and thereby may also lead to improvements of the skin conditions in psoriasis. How can these parameters effectively be modified?

One known approach to modify the Cav-1 content in the plasma membrane (and thus also the release and uptake of the vesicles that constitute the intercellular communication axis) is the application of shear stress to cells. The application of laminar shear stress on the timescale of at least several hours provided a significant increase of the density of caveolae in endothelial cells, even by very low shear values of about 1 Pa both in vitro^84^ and in vivo.^85^ On the other hand, short-time (<2 min) application of an acute mechanical stress induced by osmotic swelling or by uniaxial stretching results in a rapid disassembly of caveolae and in an increase of non-clustered caveolins within the plasma membrane.^2^

Since caveolae are linked to the actin cytoskeleton, a modification of the caveolae density in the plasma membrane must be connected with a redistribution of the cytoskeleton components in the stress field. This makes the effect of mechanical stress on caveolae not only time-, but also frequency- and strain-dependent. Application of a transient stretch to the cells can cause not only the long-known stiffening of their cytoskeleton, but also its softening and fluidization.^86,87^ Fluidization of the cytoskeleton is strongly dependent on the strain (relative deformation) amplitude, and the temporal behavior of such a system is also strain-rate dependent.^88^

The application of low-frequency (about 1 Hz) mechanical forces to the cells can effectively fluidize the cytoskeleton and modify the microdomain structures of the plasma membrane. The application of mechanical forces at frequencies of 1 MHz will reduce the strain needed for fluidization of the cytoskeleton to about 10^–5^.^89^ Such a behavior is typical for ultrasound waves where the amplitudes of the particles’ displacement in the medium is inversely dependent on the frequency. Moreover, as it was shown very recently, the application of higher ultrasound pressures and higher ultrasound frequencies induces higher levels of strain in the cells causing stronger fluidization of their cytoskeleton structure.^90^ Generally, there should be an optimal ultrasound frequency producing the maximal mechanotransduction in cells of a given type. For example, mechanical stress in chondrocytes was optimized at 5.2 ± 0.8 MHz.^91^

This makes ultrasound waves an interesting instrument for the modulation of Cav-1 protein content in target tissues. Indeed, the application of ultrasound with a frequency of 1 MHz and intensity of 2.5 W/cm^2^ increased the expression of Cav-1 in HEp-2 cells.^92^ Moreover, inhibition of Cav-1 expression in this study promoted the phosphorylation of STAT3, demonstrating the indirect effect of ultrasound on this pathway. Application of ultrasound with a frequency of 1.875 MHz and intensity of 0.25 W/cm^2^ also demonstrated the involvement of Cav-1 in the endothelial tissue reaction,^93^ since this modification of endothelial tissue observed in wild-type animals disappeared in Cav-1^−/−^ mice.

Additional effects can be provided by application of supra-physiological temperatures to psoriatic lesions. On the one hand, application of supra-physiological temperatures should cause an increase of membrane fluidity, which can increase the uptake of pathogens by non-professional phagocytic cells.^45^ On the other hand, application of such temperatures can lead to production of endogenous HMW HA, which should promote release and transport of extracellular vesicles loaded with miRs and Cavs.^82^ Indeed, application of supra-physiological temperatures of about 42 °C in vitro significantly enhances the HA synthesis in cells.^94^ A considerable increase of mucopolysaccharide content in the dermis was observed after temperature increases of 3–5 °C upon application of radio-frequency currents to the skin.^95^ Finally, mild hyperthermia with supra-physiological temperatures can significantly increase expression of Cav-1 in different types of cells,^96,97^ whereas application of higher temperatures leads to reversible disassembling of Cav-1 or its internalization.^98^

## Discussion

Cav-1 expression is strongly reduced in psoriatic skin, and induced expression of Cav-1 leads to substantial improvement of psoriasiform dermatitis in mice. This effect was connected to the STAT3 pathway. Since the STAT3 pathway has been implicated in the pathogenesis of psoriasis,^8^ and ultrasound waves can modulate Cav-1 levels through STAT3,^92^ we believe that the application of high-frequency ultrasound waves is likely to demonstrate therapeutic effects in psoriasis.

Activation of Cav-1 expression should lead to the suppression of MMPs, such as MMP-2/MMP-9,^29^ which are both known to be involved in the development of various skin efflorescences and were determined to be important targets in the treatment of these pathological conditions.^28^

Several authors reported the effective application of ultrasound waves in the treatment of psoriasis. Ultrasound frequencies used in these studies were much higher than the frequencies of up to 3 MHz which are usually applied in physical therapy. Ultrasound with a frequency of 5.3 MHz producing dermal heating of about 42 °C was successfully applied for the treatment of chronic psoriatic plaques.^99^ Similarly, application of ultrasound with a frequency of about 8 MHz, producing temperatures of 42–44 °C at a depth of 2–3 mm beneath the skin surface, demonstrated significant improvements in palmoplantar psoriasis.^100^ Recently, ultrasound with a frequency of about 10 MHz (very high-frequency ultrasound), which can concentrate heating in the dermis and superficial WAT, was also successfully applied for the treatment of psoriasis vulgaris.^28^

The effective cytoskeletal fluidization and corresponding modification of the caveolae in plasma membranes of affected cells can be still achieved with very high ultrasound frequencies.^89^ At the same time, application of very high ultrasound frequencies exceeding 10 MHz has additional effects connected to the localization of absorbed ultrasound energy in the dermis and superficial WAT, which allows to produce the supra-physiological temperatures selectively in these target areas.

Application of mild hyperthermia to psoriatic plaques was also reported to be therapeutically effective. Comparable clinical outcomes were reported by application of topical exothermic pads,^101,102^ infrared heating,^103^ water bath hyperthermia,^104^ and broad-band light.^105^

Spatiotemporal distribution of temperatures and temperature gradients produced by ultrasound waves with frequencies of 3, 10, and 19 MHz in the skin and sWAT were recently investigated in.^106^ Application of ultrasound of 3, 10, and 19 MHz with intensity of 1 W/cm^2^ for 10 s produced a temperature rise in skin of approximately 1–1.5 °C, 5–9 °C and 8–16 °C, respectively. At the same time, ultrasound with a frequency of 19 MHz was able to produce high temperature gradients of up to 14 °C/mm on the interface skin/WAT. In other words, in contrast to ultrasound waves with frequencies under 3 MHz, ultrasound waves with a frequency over 10 MHz can not only cause the fluidization of the cytoskeleton in cells, but also induce the supra-physiological temperatures in the dermis and superficial WAT layer which are the spatial targets in psoriasis. For all these reasons, very high-frequency ultrasound waves above 10 MHz are a promising treatment modality in psoriasis.

## Conclusions

Low expression of Cav-1 in psoriatic lesions appears to be not only a marker, but also a target for this skin disease. This low expression in psoriatric lesions correlates with expression of Cav-1 in adjacent adipose tissue. Spatiotemporal distribution of Cav-1 in the skin and WAT is at least partly based on exosomal transport of this molecule between different types of cells. Endogenous or ectopically-induced overexpression of Cav-1 in the affected skin and superficial adipose tissue reduces inflammation and suppresses epidermal hyperproliferation, thereby ameliorating these two well-known hallmarks in psoriasis. Such overexpression can be induced by different pharmacological and physical factors, among them by application of mechanical stress and supra-physiological temperatures, and must be considered as an important target in treatment of psoriasis.

## Data Availability

Authors can confirm that all relevant data are included in the paper.
